# Integrated genomic analyses of lung squamous cell carcinoma for identification of a possible competitive endogenous RNA network by means of TCGA datasets

**DOI:** 10.7717/peerj.4254

**Published:** 2018-01-12

**Authors:** Pengbo Ning, Zhongxing Wu, Aoxue Hu, Xuepeng Li, Jun He, Xiaocheng Gong, Yuqiong Xia, Yukui Shang, Huijie Bian

**Affiliations:** 1 School of Life Science and Technology, Xidian University, Xi’an, Shaanxi, China; 2 Engineering Research Center of Molecular and Neuro Imaging, Ministry of Education, Xi’an, Shaanxi, China; 3 Department of Cell Biology, National Translational Science Center for Molecular Medicine, Fourth Military Medical University, Xi’an, Shaanxi, China

**Keywords:** Lung squamous cell carcinoma, microRNA, ceRNA network, Overall survival, lncRNA

## Abstract

The etiology of cancer includes aberrant cellular homeostasis where a compromised RNA regulatory network is a prominent contributing factor. In particular, noncoding RNAs including microRNAs (miRNAs) and long noncoding RNAs (lncRNAs) were recently shown to play important roles in the initiation, progression, and metastasis of human cancers. Nonetheless, a mechanistic understanding of noncoding RNA functions in lung squamous cell carcinoma (LUSC) is lacking. To fill this critical gap in knowledge, we obtained mRNA, miRNA, and lncRNA expression data on patients with LUSC from the updated Cancer Genome Atlas (TCGA) database (2016). We successfully identified 3,366 mRNAs, 79 miRNAs, and 151 lncRNAs as key contributing factors of a high risk of LUSC. Furthermore, we hypothesized that the lncRNA–miRNA–mRNA regulatory axis positively correlates with LUSC and constructed a competitive endogenous RNA (ceRNA) network of LUSC by targeting interrelations with significantly aberrant expression data between miRNA and mRNA or lncRNA. Six ceRNAs (PLAU, miR-31-5p, miR-455-3p, FAM83A-AS1, MIR31HG, and MIR99AHG) significantly correlated with survival (*P* < 0.05). Finally, real-time quantitative PCR analysis showed that PLAU is significantly upregulated in SK-MES-1 cells compared with 16-BBE-T cells. Taken together, our findings represent new knowledge for a better understanding the ceRNA network in LUSC biology and pave the way to improved diagnosis and prognosis of LUSC.

## Introduction

Lung cancer is the most prevalent cancer worldwide, being the top cause of cancer-related deaths in the world, including China (Chen et al., 2016). Lung cancer has two major histological types: small cell lung cancer and non–small cell lung cancer; the latter is mostly subdivided into lung adenocarcinomas and lung squamous cell carcinomas (LUSC) (Campbell et al., 2016). LUSC is highly malignant and accounts for over 400,000 new cases worldwide each year (Gandara et al., 2015). Nonetheless, it still has a poor cure rate, with the 5-year survival rate among patients with clinical LUSC stages II to IV ranging from 40% to less than 5% (Tanoue & Detterbeck, 2009). This situation is largely due to a lack of knowledge about its molecular pathogenesis, and for this reason, little effective targeted therapy for LUSC is currently approved.

The fundamental reason for cancer initiation is anomalous and disordered regulation of the homeostasis of a cellular microenvironment, leading to tumor development from healthy cells via evasion of growth suppressors, via resistance to cell death mechanisms, and through activation of invasive and metastatic characteristics (Hanahan & Weinberg, 2011). Aberrations in the RNA regulatory network are a key factor leading to the malignant transformation of healthy cells, and include signaling pathway affecting mRNA transcription levels as well as noncoding gene regulation at the post-transcriptional level (Gupta et al., 2010; Rokavec, Horst & Hermeking, 2017). Multiple studies have confirmed that genes of microRNAs (miRNAs) are deeply involved in the development and metastasis of malignant tumors and perform important functions as oncogenes or tumor suppressor genes (Ma et al., 2010; Liu et al., 2017). In comparison with miRNAs, research on long noncoding RNAs (lncRNAs) is rudimentary and shows that more diverse and complex mechanisms are involved in tumor regulation (Prensner & Chinnaiyan, 2011).

One of the latest theories about RNA regulation—competitive endogenous RNA (ceRNA)—postulates that miRNAs have been highly conserved in the course of evolution and that a miRNA can target the miRNA response elements (MREs) in the 3′ untranslated region of mRNA for identification and to induce assembly of the gene silencing complex and to eventually control the activity of multiple target genes (Wang et al., 2016; Tay, Rinn & Pandolfi, 2014). Therefore, identical MREs will result in competition among transcriptional mechanisms of different genes for the same miRNAs, thus forming a complex RNA regulation network, which will ultimately affect cell fate (Salmena et al., 2011). For example, lncRNA can have a “sponge effect” on miRNAs, thereby weakening the direct impact of miRNAs on mRNAs (Wang et al., 2010).

On the basis of a composite profile of 178 LUSC cases, a research team from the Cancer Genome Atlas Research Network reported a comprehensive analysis of the genomic and epigenomic landscape of LUSC and identified statistically significant genes altered in tumors, e.g., TP53, CDKN2A, and RB1 (Cancer Genome Atlas Research Network, 2012). This analysis offers new avenues of research into potential therapeutic targets in LUSC. On the other hand, there is still a lack of studies on noncoding RNA in LUSC with a large sample size, aimed at identifying miRNA or lncRNA biomarkers or their key roles in post-transcriptional regulation. Considering the ceRNA theory, we assume that there is competition between gene transcripts with identical MREs, which serve as a target for the same miRNA, thus forming a complex RNA regulatory network. In this study, we used RNA sequence results from 504 samples of LUSC tumor tissues and 46 samples of adjacent non-tumorous lung tissues from The Cancer Genome Atlas (TCGA), which provides an RNA sequence platform including mRNA, miRNA, and lncRNA data on LUSC. Using this large-scale sequence database, we explored LUSC-specific miRNA and lncRNA expression profiles. Particularly, we constructed a ceRNA coexpression network of LUSC by target analysis, which can help clarify the functions of noncoding RNAs in LUSC.

## Materials and Methods

### Computational analysis of RNA sequence data for clinical variables of LUSC

We employed all the available RNA expression profile data (level 3) for 504 LUSC tumors and 46 samples of adjacent non-tumorous lung tissues from TCGA in 2016. TCGA data are divided into three levels, and level 3 data are public, containing high-level summaries such as expression quantifications of genes (Zhu, Qiu & Ji, 2014; Alcaraz et al., 2017). All of datasets in the present work were obtained in their processed level 3 form. The exclusion criteria were the following: (i) clinical stage not clear (four cases), (ii) cancer diagnosis other than LUSC (71 cases), and (iii) overall survival longer than 2,000 days (54 cases). In total, 375 LUSC samples were included in this study. Among these 375 LUSC datasets from TCGA, the available tumor data were subdivided into an early-stage group (Stages I, IA, and IB according to histopathological analysis), mid-stage group (Stages II, IIA, and IIB), and late-stage group (Stages III, IIIA, IIIB, and IV), and the 46 samples of adjacent non-tumorous lung tissues were designated as the control group. For each group, the data distribution of the obtained RNA sequencing data for both small RNAs and long RNAs is shown in Table 1. This study was conducted in full compliance with the publication guidelines of TCGA. The data were retrieved from the database of TCGA, and therefore approval of an ethics committee was not required. Clinical characteristics of patients in this study are shown in Table S1 in the Supplement. Their corresponding mRNA, miRNA, and lncRNA expressions including associated adjacent non-tumorous lung tissue samples were listed in Tables S2–S4, respectively.

**Table 1:** Sample distribution of lung squamous carcinoma in the RNA sequencing data from TCGA.

	Control group	Early-stage group	Mid-stage group	Late-stage group
All samples	46	175	122	78
miRNA sequencing samples	36	164	119	73
mRNA and lncRNA sequencing samples	40	175	122	78

At the next step, we compared the differentially expressed mRNAs, miRNAs, and lncRNAs: the early-stage group vs. control group, mid-stage group vs. control group, and late-stage group vs. control group. In this study, significant differences in gene expression were defined by two criteria: false discovery rate (FDR ≤ 0.05 and fold change ≥3). A flow chart for bioinformatics analysis was depicted in Fig. S1.

### Gene ontology and pathway analysis

Statistically significant differentially expressed mRNAs were examined in the Gene Ontology (GO) database (http://www.geneontology.org), where significantly enriched GO terms were identified to analyze their biological function (Gene Ontology Consortium, 2006). With the help of Kyoto Encyclopedia of Genes and Genomes (KEGG; http://www.kegg.jp/) (Kanehisa et al., 2008; Draghici et al., 2007), the pathway enrichment analysis uncovered the metabolic pathways and signal transduction pathways significantly enriched in LUSC (*P* ≤ 0.05 and FDR ≤ 0.05) (Benjamini & Hochberg, 1995). Both upregulated and downregulated genes were analyzed.

### Seed match analysis and construction of the ceRNA network

The miRNA seed sequences were determined by mapping the TCGA miRNA identifiers to miRBase (www.miRBase.org, release_21). The mRNA target genes of differentially expressed miRNAs in this study were predicted using miRanda (http://www.microrna.org/) and Targetscan (http://www.targetscan.org/). The miRanda (http://www.microrna.org/) was also applied to predict the lncRNAs targeted by miRNAs. The corresponding miRNA–mRNA and miRNA–lncRNA paired libraries were listed in Tables S5 and S6, respectively.

According to the theory that lncRNAs can act as a miRNA sponge by sequestering and binding them to further regulate mRNA activity, the miRNAs negatively regulated by the competing expression levels of lncRNAs and mRNAs were selected to construct a lncRNA–miRNA–mRNA ceRNA network (upregulated or downregulated fold change ≥3, FDR ≤ 0.05, and *P* < 0.05) (Li et al., 2016). Cytoscape v3.0 was used to construct the interactive and visual ceRNA network.

### Clinical features of key members of the ceRNA network

Using the obtained ceRNA network, we then analyzed the clinical features for assessment of patients’ outcomes. The Cox proportional hazards regression model was employed to analyze the association among the mRNAs, miRNAs, and lncRNAs from the ceRNA network and LUSC patient survival periods obtained from TCGA. Statistically significant mRNAs, miRNAs, and lncRNAs affecting the survival period (*P* < 0.05) were then determined by the Cox regression univariate analysis to subsequently construct the Kaplan–Meier survival curve for patients with LUSC.

### Cell culture

Human lung squamous cell carcinoma SK-MES-1 cells were purchased from the Type Culture Collection of the Chinese Academy of Sciences (Shanghai, China). Human bronchial epithelial 16-HBE-T cells were acquired from MssBio Co., Ltd. (Guangzhou, China). SK-MES-1 cells were cultured in the Minimum Essential Medium (Grand Island, New York, NY, USA) supplemented with 10% (v/v) of fetal bovine serum (FBS), Glutamax, nonessential amino acids, and a sodium pyruvate solution (0.1 mol/L). 16-HBE-Tcells were cultured in the RPMI-1640 medium (Grand Island, New York, NY, USA) supplemented with 10% of FBS. All the cell lines were grown in a humidified incubator (5% CO_2_) at 37 °C.

### RNA extraction and quantitative PCR

Total RNA was extracted from the cells using the TRIzol Reagent (Invitrogen, Carlsbad, CA, USA). Single-strand cDNA was synthesized from 1 μg of total RNA using the Prime-ScriptTM Reagent Kit with gDNA Eraser (Takara, Dalian, China). Real-time quantitative PCR (RT-qPCR) primers were purchased from the Beijing Genomics Institute. The primers were as follows: PLAU sense, 5′-TCACCACCAAAATGCTGTGT-3′, and antisense, 5′-CCAGCTCACAATTCCAGTCA-3′ (Xu et al., 2015). The qPCR was conducted on a 7300 Real-Time PCR system (Applied Biosystems, Life Technologies, Singapore). The following cycling conditions were applied: 95 °C for 5 min, followed by 40 cycles of 95 °C for 20 s and 60 °C for 30 s. For each sample, qPCR assays were conducted in triplicate in a 10 μl reaction volume. β-Actin served as an internal control to normalize the expression of PLAU. The 2^−ΔΔCt^ method was employed to calculate the relative expression of PLAU mRNA (Livak & Schmittgen, 2001).

### Statistical analysis

These analyses were performed using the SPSS 21.0 software. All the data are presented as mean ± SD. The statistical analysis involved Student’s *t* test to compare two groups, and ANOVA was conducted for multiple comparisons. Differences with *P*-value <0.05 were considered statistically significant. The significance level was set to 0.05 as a default to control the FDR. The univariate Cox proportional hazards regression was applied to determine the ceRNAs correlating with overall survival.

## Results

### Differentially expressed genes and their GO and pathway analysis

**Figure 1:**
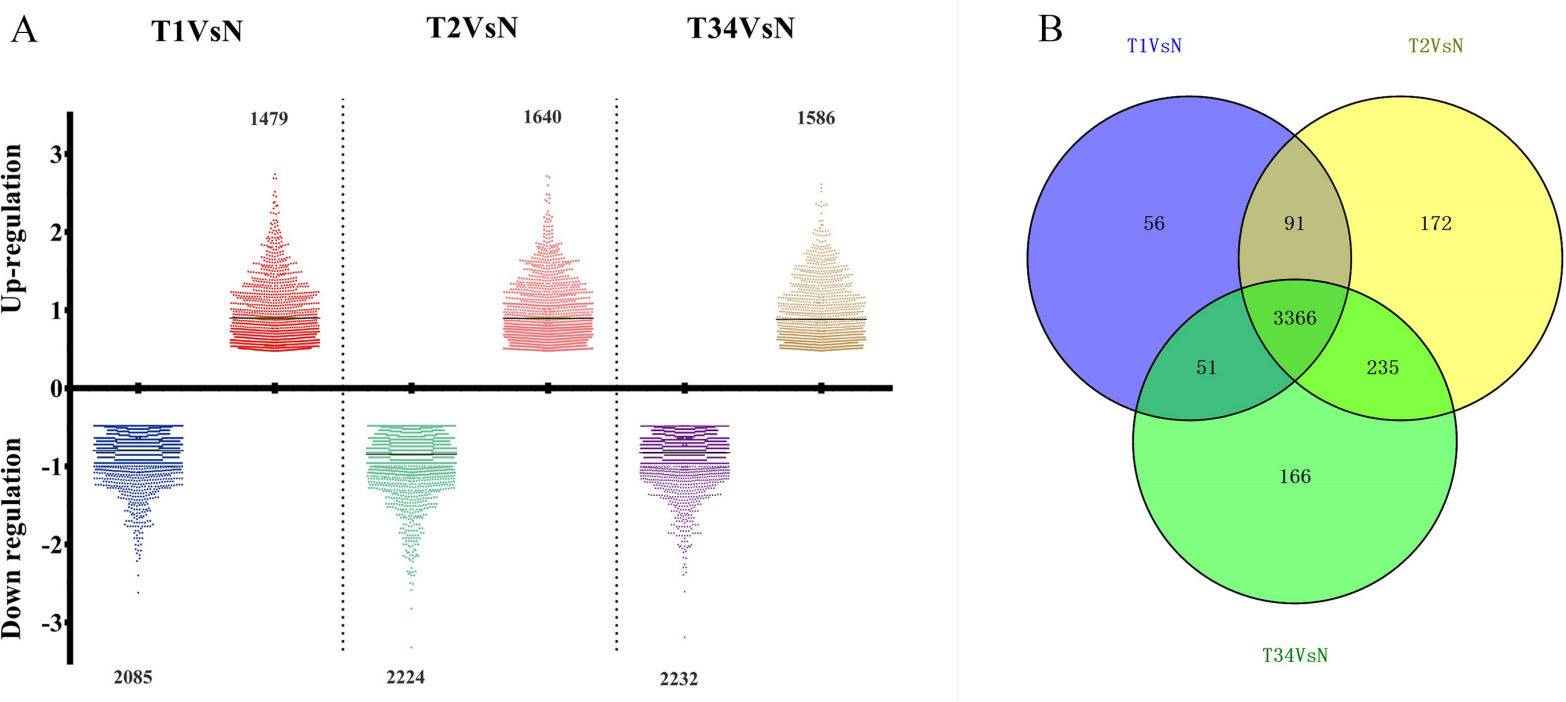
The number of differentially expressed genes obtained from the TCGA data among LUSC groups and controls. (A) Three thousand, five hundred sixty-four mRNAs were differentially expressed between the early-stage group “T1” and control group “N,” 3,864 mRNAs were differentially expressed between the mid-stage group “T2” and control group “N,” and 3,818 mRNAs were differentially expressed between the late-stage group “T3” and control group “N.” (B) Venn diagrams represent comparison of known and novel miRNAs among three data sets. The number marked in the overlapping areas indicates the common miRNAs. The control “N” represents adjacent non-tumor tissues.

**Figure 2:**
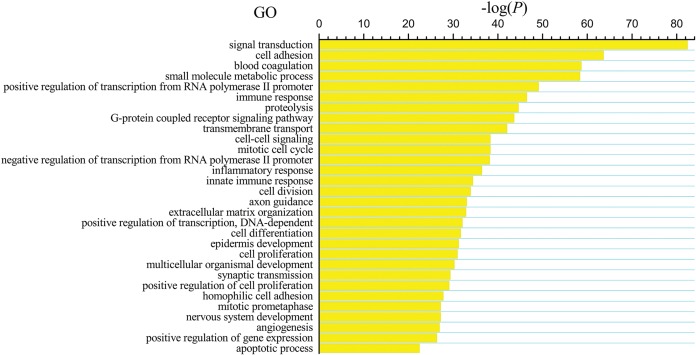
Key gene ontology (GO) terms of differentially expressed intersection mRNAs (the bar plot shows the enrichment scores of the significant enrichment GO terms).

The differentially expressed genes obtained from the TCGA data were analyzed among LUSC groups and controls. We found 3,564 mRNAs to be differentially expressed between the early-stage group and control group, 3,864 mRNAs were differentially expressed between the mid-stage group and control group, and 3,818 mRNAs were differentially expressed between the late-stage group and control group (Fig. 1A). All significantly expressed genes are listed in Table S7. A Venn diagram (Fig. 1B) was constructed to illustrate the distribution of genes whose expression was common among groups or unique to each LUSC group. In total, 3,366 common differentially expressed genes were found in each library when compared with the control. Finally, the 3,366 differentially expressed genes were further analyzed using the GO database (http://www.geneontology.org). Enrichment of these differentially expressed genes represents a measure of significance of a function; for instance, the most enriched GO was “signal transduction” and other significant GO terms included “cell adhesion,” “blood coagulation,” and “immune response” (Fig. 2). These data provided a definitive functional description of the genes differentially expressed in LUSC.

**Figure 3:**
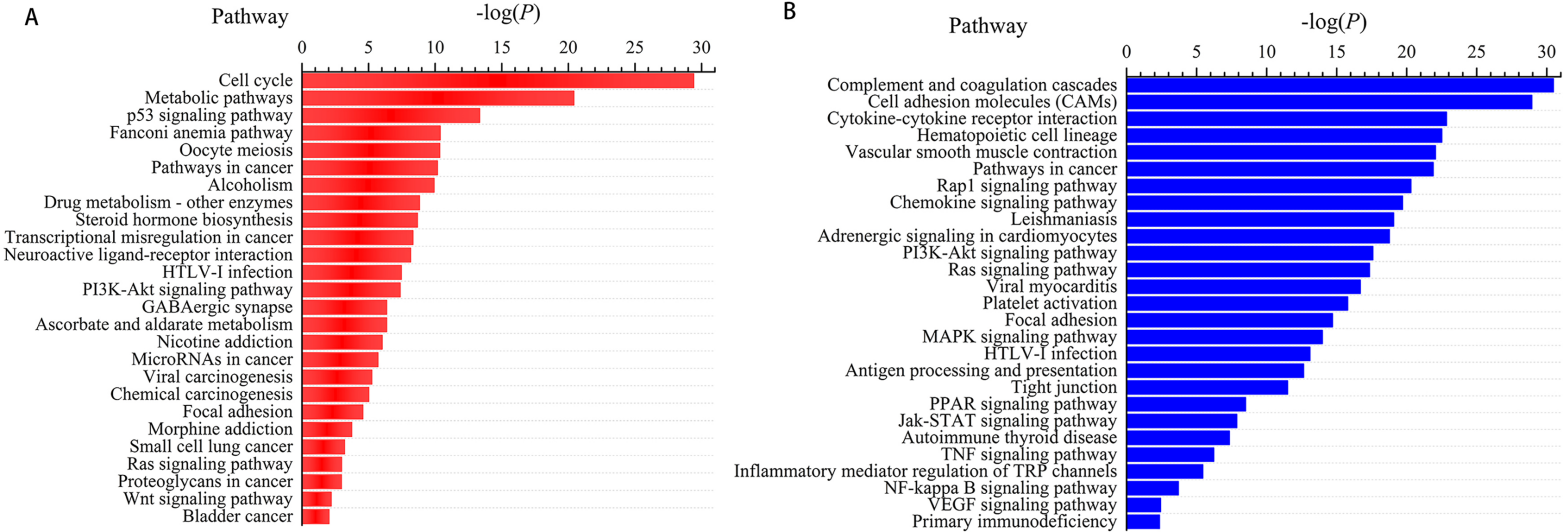
Pathways enrichment analysis for LUSC groups versus controls (the bar plot shows the enrichment scores of the significant enrichment pathways). (A) Key pathways in upregulated, differentially expressed genes; (B) key pathways in downregulated, differentially expressed genes.

We next subjected the 3,366 differentially expressed genes to pathway analysis, where the most enriched network corresponding to the upregulated transcripts turned out to be “cell cycle” and the most enriched network corresponding to the downregulated transcripts was “complement and coagulation cascades.” Moreover, pathway analysis showed that the “p53 signaling pathway,” “Fanconi anemia pathway,” “transcriptional misregulation in cancer,” “microRNAs in cancer,” and “small cell lung cancer” correspond to the upregulated transcripts (Fig. 3A) whereas “cell adhesion molecules (CAMs),” “hematopoietic cell lineage,” “PI3K-Akt signaling pathway,” “Ras signaling pathway,” and “Jak-STAT signaling pathway” correspond to the downregulated transcripts (Fig. 3B). In addition, “pathways in cancer” was identified as the significant gene category involved in the development of LUSC corresponding to both the upregulated and downregulated transcripts (Fig. 3).

### Cancer-specific miRNAs and lncRNAs in LUSC and their ceRNA network

**Figure 4:**
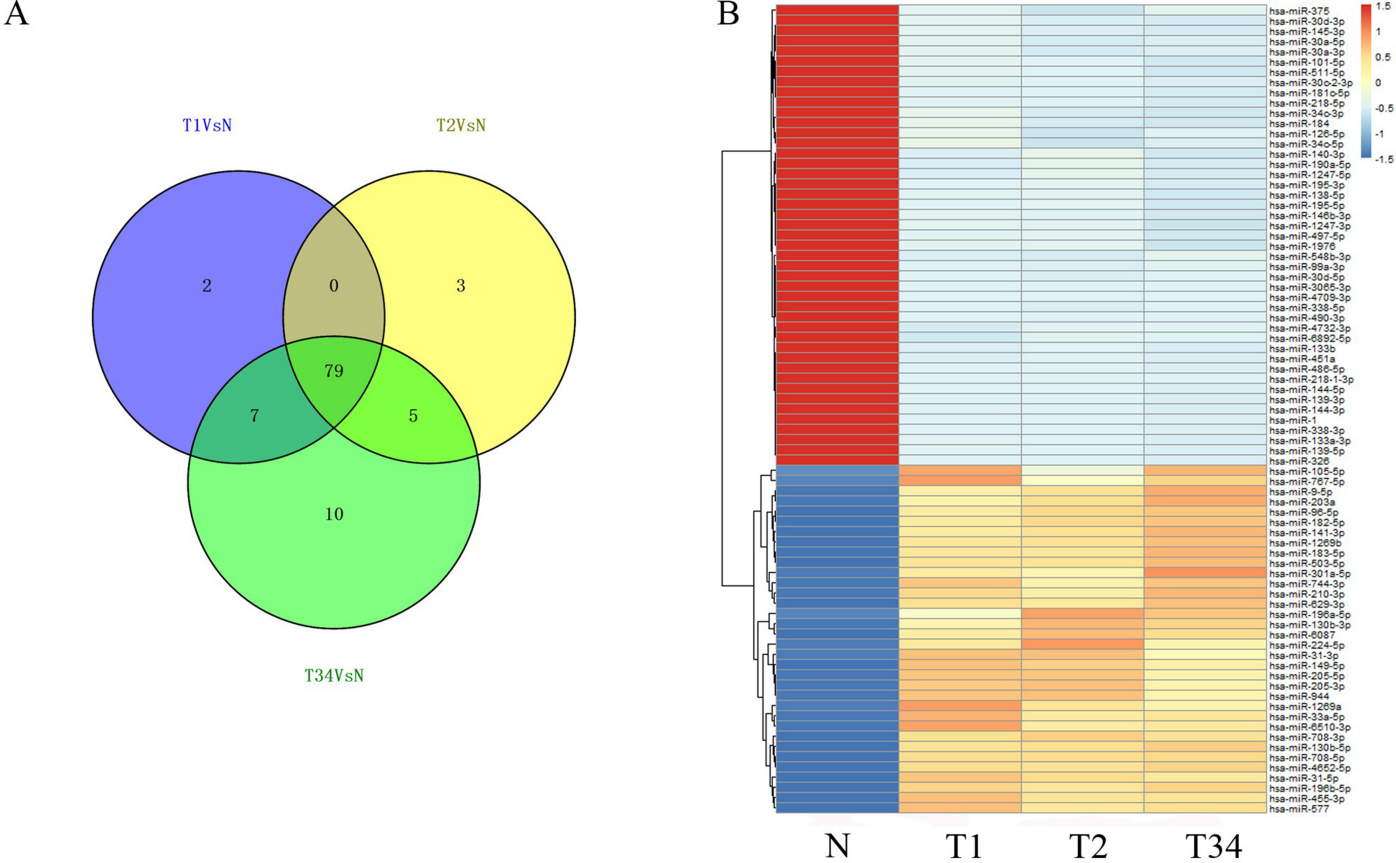
The differentially expressed miRNAs obtained from the TCGA data among LUSC groups and controls. (A) Venn diagrams represent comparison of three data sets, and the number marked in the overlapping areas indicates the 79 common miRNAs; (B) list of differentially expressed 79 mRNAs between LUSC groups and controls. T1, T2, and T3 represent the early-stage group, the mid-stage group and the late-stage group, respectively. The control (N) represents adjacent non-tumor tissues.

We then identified the miRNAs and lncRNAs differentially expressed between the LUSC tumor tissues and the adjacent non-tumorous tissues from the TCGA database (absolute fold change >2, *P* < 0.05). In the analysis of differentially expressed miRNAs, 88 miRNAs were found to be differentially expressed between the early-stage group and control group, 87 miRNAs were differentially expressed between the mid-stage group and control group, and 101 were differentially expressed between the late-stage group and control group. Figure 4A shows the distribution of miRNAs expressed in all three libraries, and 79 miRNAs that were coexpressed in the three libraries (Fig. 4B) were selected to build the ceRNA network.

**Figure 5:**
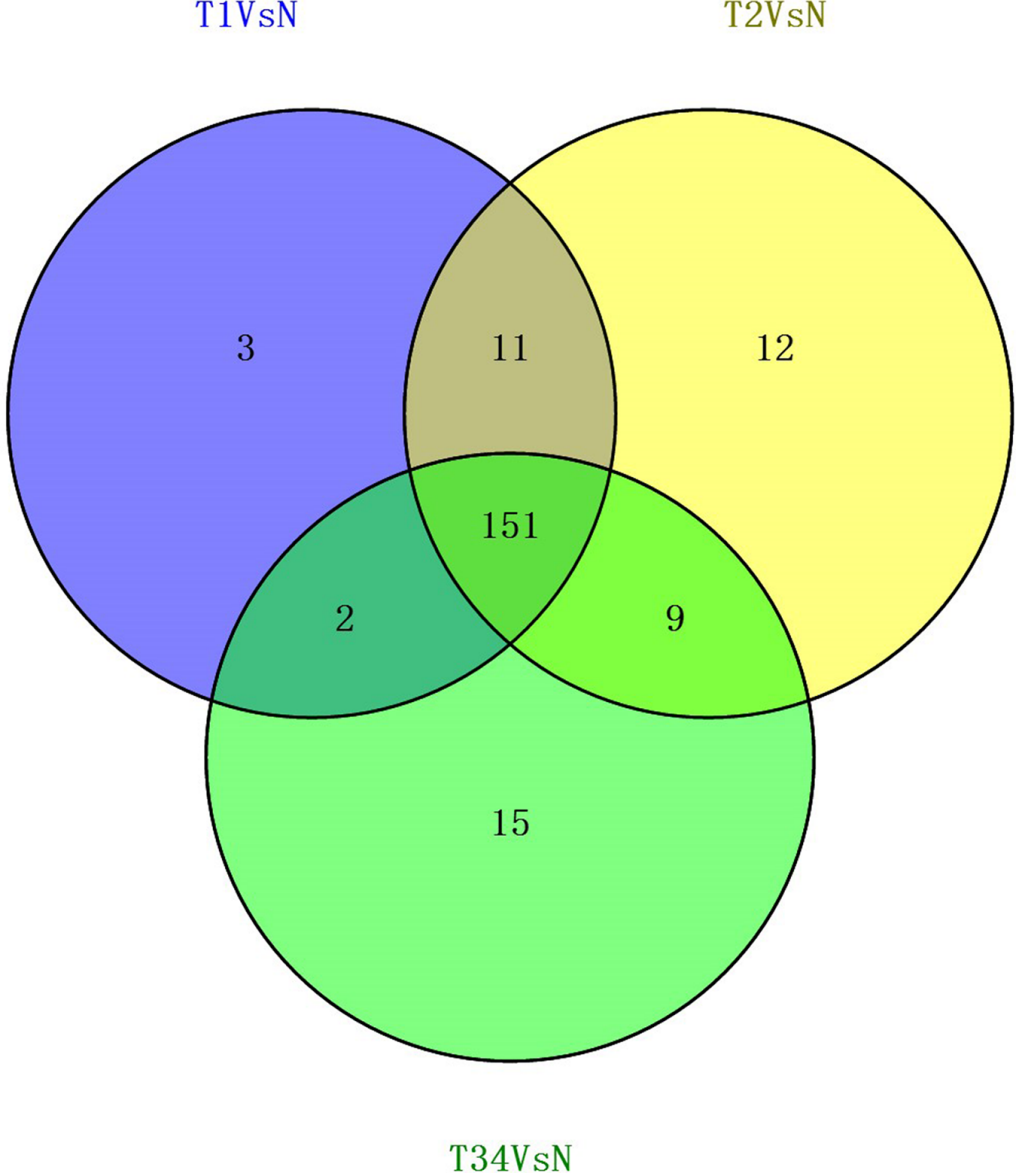
Venn diagrams showed the number of differentially expressed lncRNAs obtained from the TCGA data among LUSC groups and controls.

**Figure 6:**
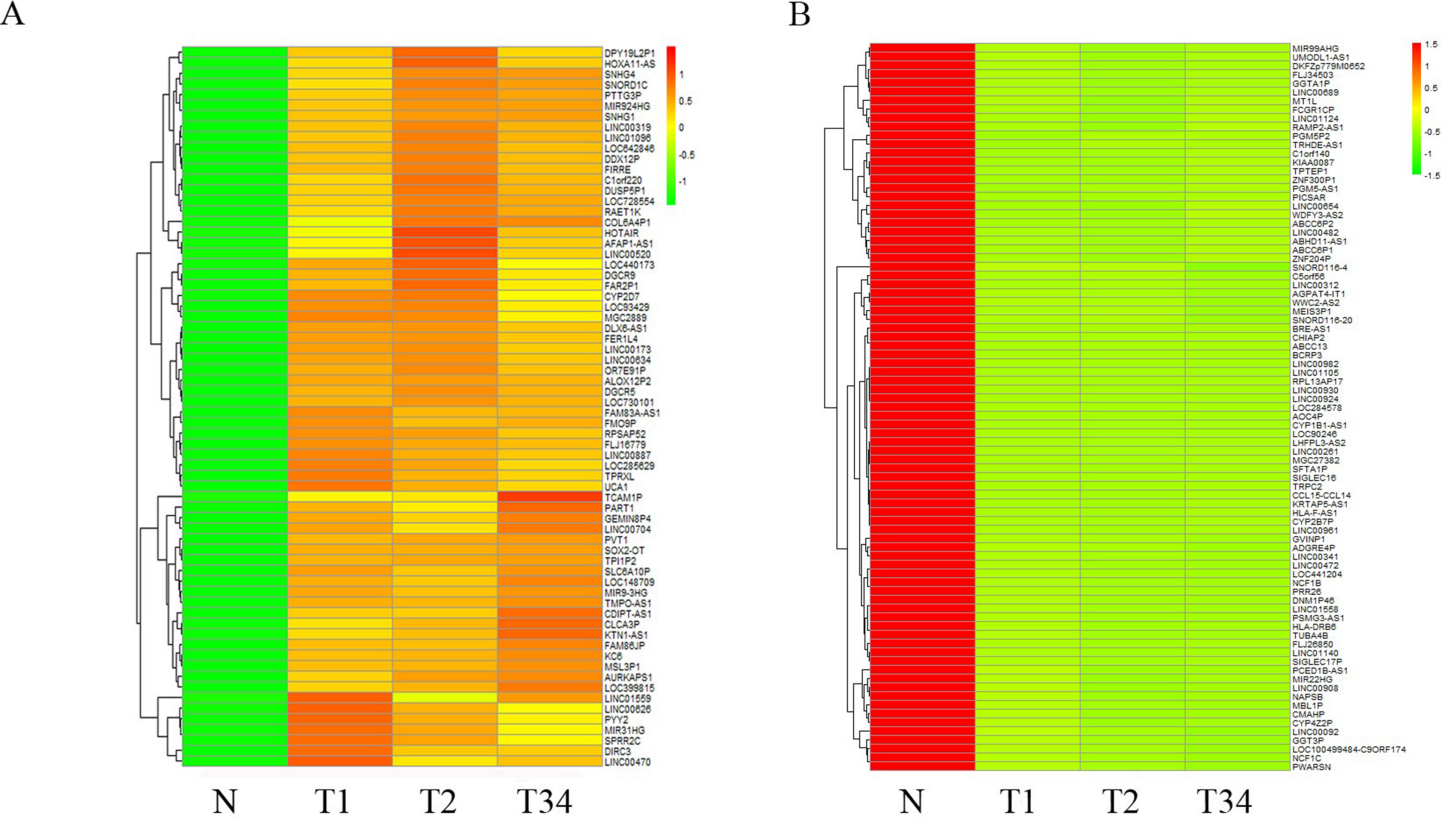
List of differentially expressed 151 lncRNAs among LUSC groups and controls. (A) Key pathways in 68 upregulated expressed lncRNAs; (B) 83 downregulated expressed lncRNAs. T1, T2, and T3 represent the early-stage group, the mid-stage group and the late-stage group, respectively. The control (N) represents adjacent non-tumor tissues.

We also studied the distribution of lncRNAs to illustrate which lncRNAs are differentially expressed between the LUSC tumor tissues and adjacent non-tumorous tissues. We found that 167, 183, and 177 lncRNAs were differentially expressed between the groups “early-stage” and “control,” between the groups “mid-stage” and control, and between the groups “late-stage” and control (Fig. 5), respectively. Finally, 151 lncRNAs (68 upregulated and 83 downregulated) coexpressed in the three libraries were selected to build the ceRNA network (Fig. 6).

**Figure 7:**
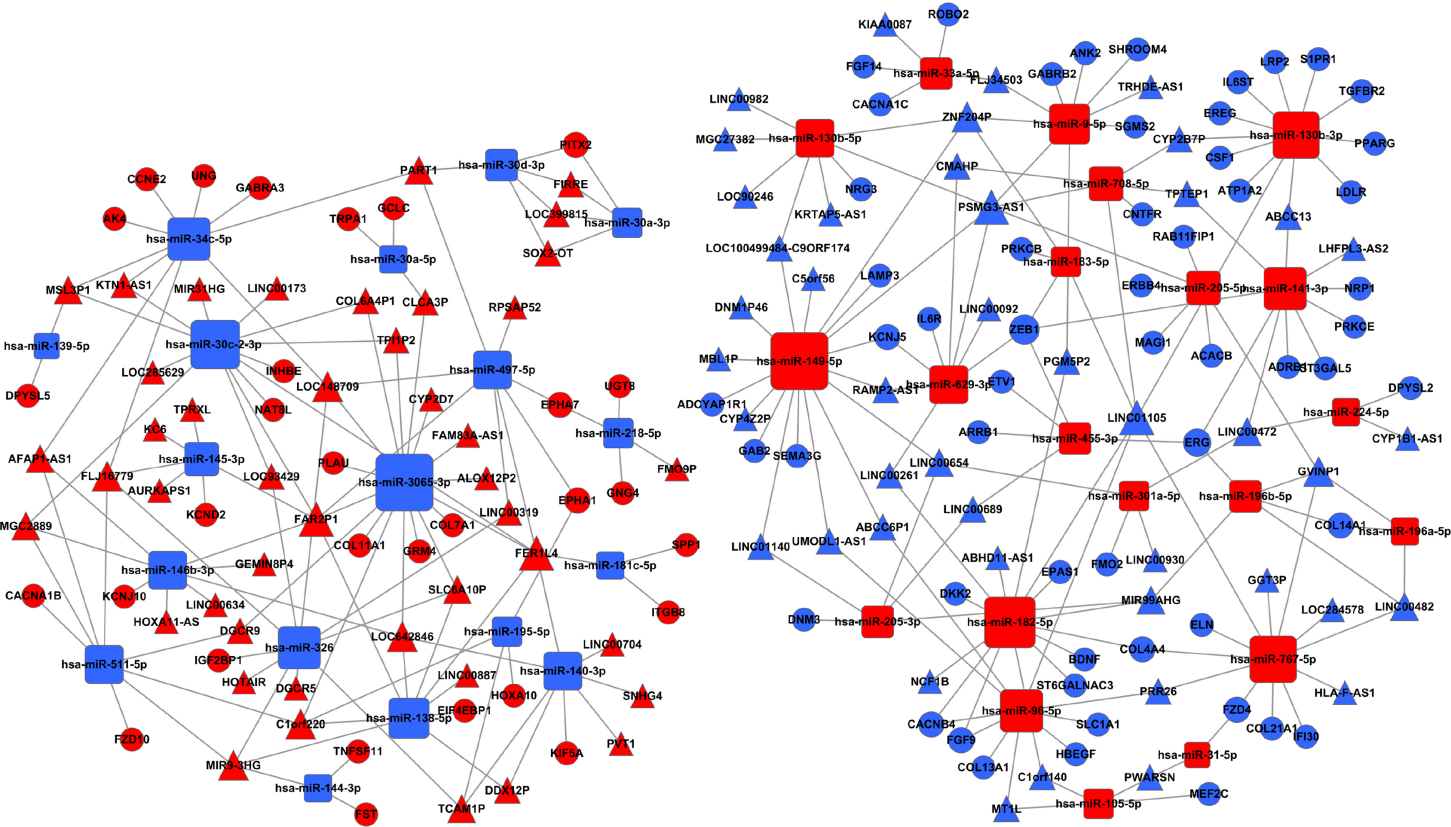
The ceRNA network of lncRNA–miRNA–mRNA in LUSC. The red represents the upregulated, and the blue represents the downregulated. Diamonds represent miRNAs, balls represent mRNAs, and triangles represent lncRNAs.

Further, we search for mRNAs targeted by miRNAs from the dataset between the Figs. 1 and 4B, and also predicted miRNAs targeted lncRNAs from the dataset between the Figs. 4B and 6. The ceRNA network of LUSC was built in Fig. 7 through the mRNA–miRNA–lncRNA of negative regulation. 86 mRNAs, 39 miRNAs and 88 lncRNAs were integrated into the ceRNA network of LUSC (Fig. 7).

### Key ceRNAs and their associated clinical features

**Figure 8:**
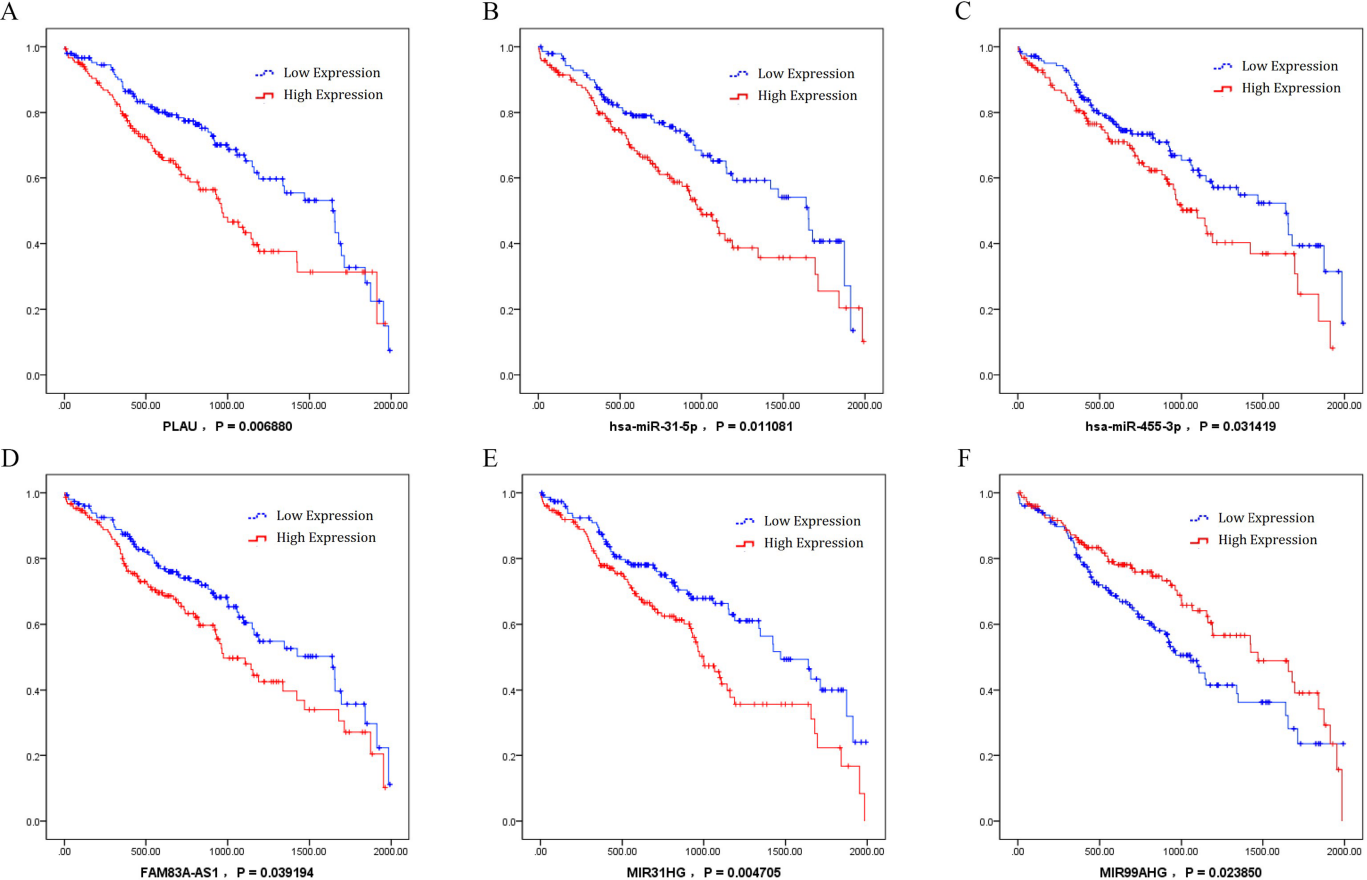
Kaplan–Meier survival curves for six ceRNA (PLAU, miR-31-5p, miR-455-3p, FAM83A-AS1, MIR31HG, and MIR99AHG) associated with overall survival. Horizontal axis: overall survival time, days; Vertical axis: survival function. (A) PLAU was negatively associated with overall survival; (B) miR-31-5p was negatively associated with overall survival; (C) miR-455-3p was negatively associated with overall survival; (D) FAM83A-AS1 was negatively associated with overall survival; (E) MIR31HG was negatively associated with overall survival; (F) MIR99AHG positively correlated with overall survival.

By analysis of the association between key ceRNAs and LUSC patients’ survival periods, ceRNAs were chosen according to the bioinformatics analysis and the ceRNA network to analyze the effects significant for survival (*P* value less than 0.05) in order to identify the specific ceRNAs with prognostic characteristics. Among the differentially expressed ceRNAs, one mRNA (*PLAU*), two miRNAs (miR-31-5p and miR-455-3p), and three lncRNAs (FAM83A-AS1, MIR31HG, and MIR99AHG) were found to be associated with the overall survival of patients with LUSC by univariate Cox regression analysis. Kaplan–Meier survival curves indicated that the lncRNA MIR99AHG positively correlated with overall survival, whereas *PLAU*, miR-31-5p, miR-455-3p, FAM83A-AS1, and MIR31HG were negatively associated with overall survival (Fig. 8).

### Characteristics of PLAU in LUSC

**Figure 9:**
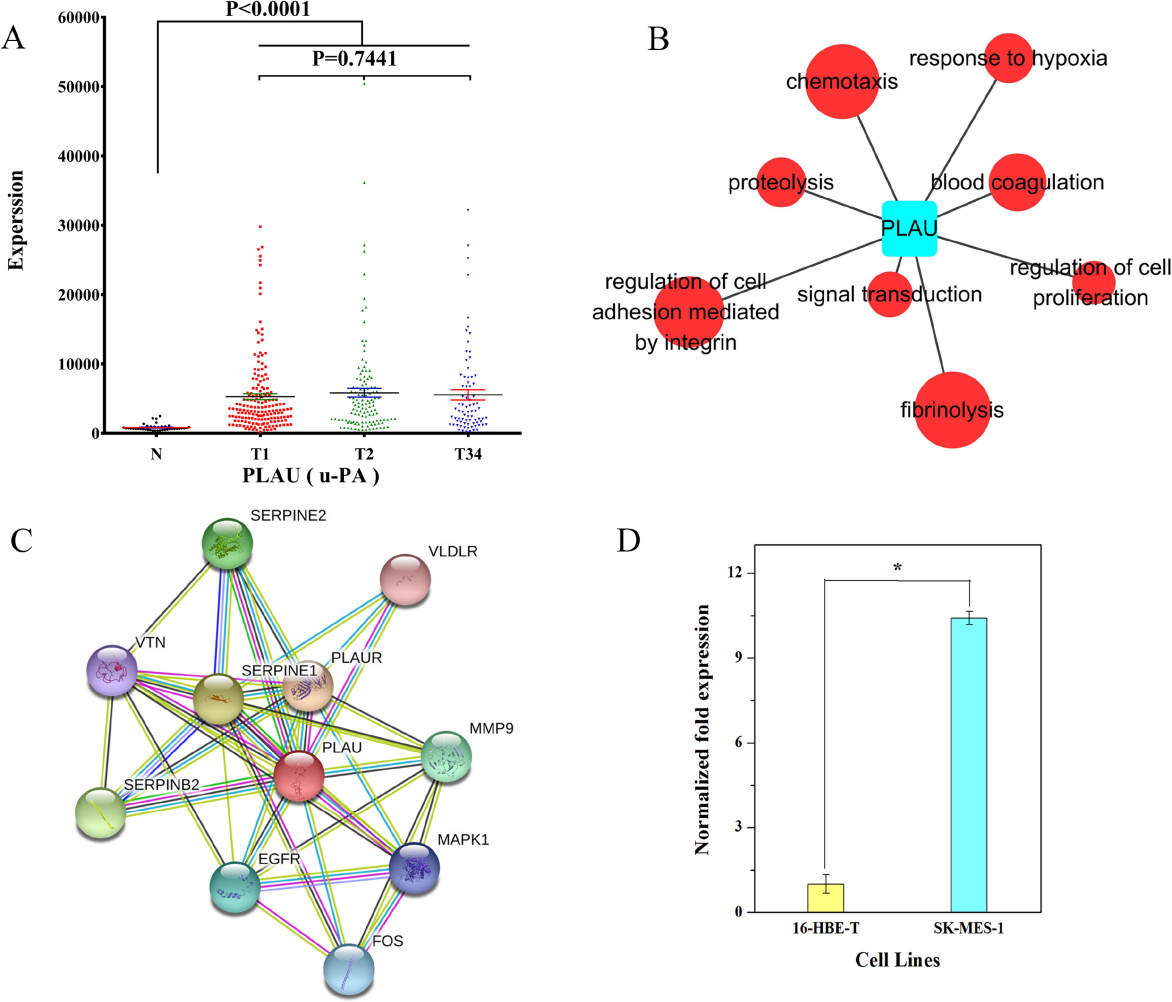
Characteristics of PLAU in LUSC. (A) PLAU was significantly upregulated at the early, middle, and late stages of LUSC relative to the adjacent non-tumorous tissues; (B) GO analysis showed anomalous expression of PLAU was related to aberrant regulation of gene function groups such as chemotaxis, proteolysis, and fibrinolysis; (C) protein–protein interaction analysis of urokinase plasminogen activator (uPA, a.k.a. PLAU); (D) the expression of PLAU in SK-MES-1 cells compared with 16-HBE-T cells. *P < 0.05.

We examined the expression data on *PLAU* in different groups of LUSC. The result showed that *PLAU* was significantly upregulated at the early, middle, and late stage of LUSC relative to the adjacent non-tumorous tissues (Fig. 9A). According to GO analysis, anomalous expression of *PLAU* is related to aberrant regulation of gene function groups such as pulmonary squamous cell carcinoma, proteolysis, and fibrinolysis (Fig. 9B). Protein–protein interaction analysis revealed that urokinase plasminogen activator (uPA, a.k.a. *PLAU*) can interact with several proteins including serine protease inhibitor1 (SERPINE1), SERPINE2, plasminogen activator inhibitor2 (SERPINB2), vitronectin (VTN), plasminogen activator receptor (PLAUR), very low density lipoprotein receptor (VLDLR), matrix metallopeptidase 9 (MMP9), epidermal growth factor receptor (EGFR), FOS protein, and mitogen-activated protein kinase 1 (MAPK1; Fig. 9C).

We also evaluated the expression of *PLAU* in SK-MES-1 cells compared with 16-HBE-T cells. As shown in Fig. 9D, uPA transcript levels were significantly higher in SK-MES-1 cells than in 16-HBE-T cells (up to 9.99-fold).

## Discussion

The progression and metastasis of lung cancer in the host remain an enigma, but not for a lack of effort (Reck et al., 2013). Recent technological advances in high-throughput sequencing and bioinformatics have enhanced our understanding of the genesis and characteristics of LUSC. As a publicly funded project, TCGA is intended for cataloging and discovery of the major cancer-causing genomic variations to form a comprehensive outline of the cancer genome (Tomczak, Czerwińska & Wiznerowicz, 2015). The present study updates the latest genomic information on LUSC by means of a large number of genome sequences and by integrated multidimension analyses of TCGA datasets from the year 2016. Our analysis suggests that LUSC involves dynamic changes in the genome, and genetic changes strongly correlating with the tumor appear as altered gene expression profiles. For example, one finding shows that TP63, TP73, and SOX2 expression in tumor tissues of early, middle, and late stages is higher than that in the adjacent non-tumorous tissues. This observation suggests that aberrant amplification of SOX2 drives the overexpression of TP63 and TP73 as excellent biomarkers contributing to LUSC progression. These findings are consistent with those of another study (Moll & Slade, 2004), and additional valuable driver genes should be identified after further research. Highlighting the deregulation of mRNAs by GO and pathway analyses, this study suggests that LUSC cell functions have significant differences from those in healthy cells, and that many genes significantly differentially expressed in LUSC are enriched in cancer-related pathways such as “p53 signaling pathway” and “Fanconi anemia pathway.” Bretz et al. (2016) also demonstrated that TP63, a member of the p53 gene family, directly activates the Fanconi anemia pathway in LUSC; therefore, targeting TP63 may not only hamper squamous cell carcinomas progression but also sensitize tumors to cisplatin treatment.

Recent advances revealed that miRNA genes can be considered novel oncogenes or tumor suppressor genes involved in the initiation, progression, invasiveness, and metastasis of tumors (Liu et al., 2017). In the present study, 79 miRNAs were found to be significantly differentially expressed at the early, middle, and late stages of LUSC in comparison with adjacent non-tumorous tissues, and some of them have been reported to function in the mechanisms of tumor metastasis. Furthermore, recent studies highlighted the role of lncRNAs in carcinogenesis and suggest that this group of genes may serve as biomarkers of cancer (Xie, Ma & Zhou, 2013; Li et al., 2017). Among the 151 lncRNAs uncovered in this study, lncRNA TUBA4B has been reported as a predictor of poor prognosis and a regulator of cell proliferation in non-small cell lung cancer (Chen et al., 2017). Amplification of PVT-1 is associated with poor prognosis via inhibition of apoptosis in colorectal cancer (Takahashi et al., 2014). Upregulated lncRNA SNHG1 contributes to the progression of non-small cell lung cancer through activation of the Wnt/β-catenin signaling pathway (Cui et al., 2017). Expression of LINC00312 negatively correlates with tumor size but positively correlates with lymph node metastases in nasopharyngeal carcinoma (Zhang et al., 2013).

Studies indicate that ceRNAs are key regulators in the communication among different RNA transcripts and participate in oncogenesis and cancer progression (Tay, Rinn & Pandolfi, 2014). lncRNA HULC (highly upregulated in liver cancer) and lncRNA PTCSC3 (papillary thyroid carcinoma susceptibility candidate 3) are two classic cases in this field, highlighting different roles of miRNA–lncRNA competitive interactions. HULC has been reported as a highly upregulated gene in the hepatocellular carcinomas compared to healthy liver tissues (Xie, Ma & Zhou, 2013). HULC may act as an endogenous “sponge” to downregulate miR-372, thus leading to translational derepression of PRKACB, sequentially inducing phosphorylation of CREB (Wang et al., 2010). Compared with lncRNA HULC, lncRNA PTCSC3 is a newly identified noncoding RNA that is dramatically downregulated in thyroid cancers and has been studied as a tumor suppressor that competes with endogenous RNA for miR-574-5p (Fan et al., 2013).

Therefore, there may be some possible interaction among lncRNA, miRNA, and mRNA in the progression and metastasis of LUSC. In the present study, ceRNA networks of LUSC were built by bioinformatic prediction and correlation analysis of data on significantly altered expression of lncRNA, miRNA, and mRNA. Furthermore, regarding the associations between cancer-specific ceRNA numbers and clinical features, we found that six ceRNAs are related to clinical features. Some of these have been implicated in cancer. MiRNA expression profiling suggests that miR-31-5p may act as a new biomarker enabling identification of patients with metastatic colorectal cancer harboring wild-type RAS with possible shorter time to progression and resistance to cetuximab therapy (Igarashi et al., 2014). MiR-455-3p was found to play a role in acquired temozolomide resistance and may be a novel therapeutic target in recurrent glioblastoma multiforme (Ujifuku et al., 2010). LncRNA MIR31HG is upregulated in pancreatic ductal adenocarcinoma and manifests oncogenic properties by influencing cell proliferation and invasiveness (Yang et al., 2016).

In the present study, Kaplan–Meier survival curves indicated that upregulated *PLAU* positively correlates with poor survival among patients with LUSC. *PLAU* is the gene encoding uPA (Su et al., 2016). Extracellular matrix degradation is a key precursor of tumor metastasis and is involved in the multistep process of tumor invasion and metastasis, including tumorstromal infiltration and invasion, angiogenesis, and transformation of the tumor microenvironment (Sidenius & Blasi, 2003; Gkretsi, Stylianou & Stylianopoulos, 2017). The anomalous expression of *PLAU* (uPA) as an oncogeneis associated with tumors encroaching on the extracellular matrix and has been reported to participate in the development and progression of a variety of cancers (Dass et al., 2008). Nevertheless, there are no reports on the association of *PLAU* with LUSC. In the present study, *PLAU* was found to be upregulated at the early, middle, and late stages of LUSC compared to the adjacent non-tumorous tissues. Moreover, upregulated *PLAU* positively correlated here with poor survival among the patients, suggesting that *PLAU* may be a new biological marker of (and/or a therapeutic target in) LUSC.

## Conclusion

Taken together, our results identify cancer-specific mRNAs and noncoding RNAs in LUSC by bioinformatics analysis of thousands of candidate RNAs and large-scale samples from the database of TCGA. Using these results, we constructed a ceRNA network and studied the significance of the expression pattern of LUSC-specific ceRNAs (*PLAU*, miR-31-5p, miR-455-3p, FAM83A-AS1, MIR31HG, and MIR99AHG) for overall survival of patients with LUSC. Moreover, we used qRT-PCR to validate the high expression of *PLAU* in SK-MES-1 cells compared with 16-HBE-T cells. Our study provides novel insights into the ceRNA network in LUSC and offers candidate biomarkers for diagnosis and prognosis.

## Supplemental Information

10.7717/peerj.4254/supp-1 Supplemental Information 1 An overall workflow of bioinformatics analysis on the identification of a possible competitive endogenous RNA network to lung squamous cell carcinoma.

10.7717/peerj.4254/supp-2 Supplemental Information 2 Clinical characteristics of patients on lung squamous cell carcinoma of TCGA in 2016.

10.7717/peerj.4254/supp-3 Supplemental Information 3 Corresponding mRNAs of clinical patients on lung squamous cell carcinoma of TCGA in 2016. (Gene ID 1-10000).

10.7717/peerj.4254/supp-4 Supplemental Information 4 Corresponding miRNAs of clinical patients on lung squamous cell carcinoma of TCGA in 2016.

10.7717/peerj.4254/supp-5 Supplemental Information 5 Corresponding lncRNAs of clinical patients on lung squamous cell carcinoma of TCGA in 2016.

10.7717/peerj.4254/supp-6 Supplemental Information 6 Targeted mRNAs to significantly expressed miRNAs from TCGA datasets of lung squamous cell carcinoma.

10.7717/peerj.4254/supp-7 Supplemental Information 7 Targeted lncRNAs to significantly expressed miRNAs from TCGA datasets of lung squamous cell carcinoma.

10.7717/peerj.4254/supp-8 Supplemental Information 8 All significantly expressed genes between lung squamous cell carcinoma groups and the control group.

## Additional Information and Declarations

### Data Availability

The following information was supplied regarding data availability: The raw data has been provided as Supplemental Dataset Files.
