# Supplementary figures and images for: Integrated genomic analyses of lung squamous cell carcinoma for identification of a possible competitive endogenous RNA network by means of TCGA datasets
Source: PeerJ. 2018 Jan 12;6:e4254. doi: 10.7717/peerj.4254 (PMC5768173; doi:10.7717/peerj.4254)

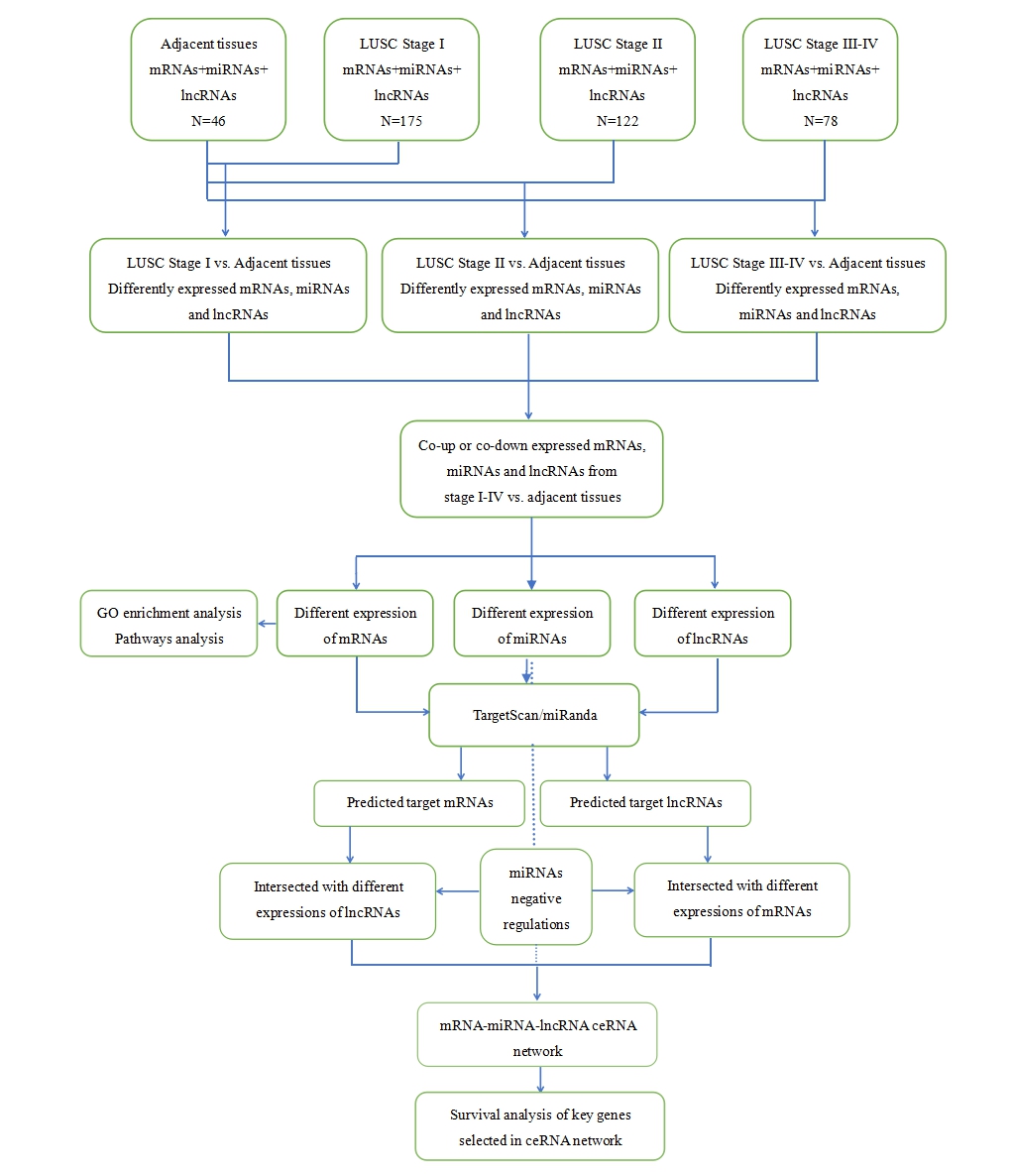

Supplement: Supplemental Information 1 [file peerj-06-4254-s001.jpg]
